# Screening and Addressing Food Insecurity at Free Clinics: A Scoping Review

**DOI:** 10.21203/rs.3.rs-6270596/v1

**Published:** 2025-05-13

**Authors:** Gautam Ramanathan, William Zak, Daiwik Munjwani, Robert Fisher, Esther May Sarino, Mary Scourboutakos

**Affiliations:** Eastern Virginia Medical School; Eastern Virginia Medical School; Eastern Virginia Medical School; Eastern Virginia Medical School; Eastern Virginia Medical School; Eastern Virginia Medical School

**Keywords:** Food Insecurity, Student Run Free Clinics, Social Determinants of Health, Food Assistance Resources

## Abstract

Food insecurity (FI) is highly prevalent amongst patients seeking care at free, student-run health clinics. This study sought to examine the existing literature to examine food insecurity screenings and interventions at free clinics across the U.S. In this review, we provide demographic information and screening statistics of food insecurity interventions, the study’s primary aim, and how it relates to the provided FI intervention, barriers that studies identified in implementing FI interventions. Eligible studies needed to implement a FI screening and intervention program in a free clinic within the United States specifically due to the unique nature of the healthcare system. Studies could address additional social determinants of health or chronic conditions, but had to include FI screenings and interventions. Of the initial database search of 958 studies, five were included for analysis. Among the studies, food insecurity was determined by the 6 item HFSS, a one question from the USDA food security survey, and a custom 12 item redcap survey. Methods of combating food insecurity included providing grocery deliveries, utilizing in clinic food pantries, providing onsite food boxes, aiding patients in accessing SNAP, WIC and food pantries in the area, and providing referrals. Few studies examined the effectiveness of implemented interventions. Future studies on implementation of programs targeting food insecurity should focus on an evaluation of the effectiveness of their program, assess the implementation of other FI screening assessments in identifying FI patients and determine the efficacy of implemented FI interventions on various patient health outcomes.

## INTRODUCTION

Food Insecurity (FI), is defined as the lack of access to affordable, nutritious food that can lead to significant health issues ranging from chronic conditions such as Type 2 Diabetes Mellitus in adults to impaired academic development in children to fetal malformations [1]. Multiple national organizations, Center for Medicare and Medicaid Services, American Academy of Pediatrics, American Diabetes Association and the USDA have screening guidelines in place to determine FI among patients that have been shown to be effective [2, 3, 4].

It has been well established that risk factors for FI among U.S families include a lower income and a lack of health insurance [5]. Furthermore, free clinics are utilized as a safety net for these populations, with one study showing that the target demographics for 75% of free clinics in the U.S are the uninsured and the low-income [6]. As a result, effective screening in free clinics can become a valuable way to address FI in at-risk populations.

There are multiple ways to screen for FI in free clinics, some which are variations of the 18-item US Household Food Security Scale (HFSS). The most notable among these is the six-item HFSS and the two-item Hunger Vital Sign questionnaire [7, 8]. While these questionnaires are both validated and reliable, some programs to address FI at free clinics have also utilized custom questionnaires [9]. Choosing the type of screening at free clinics is dependent on various factors, such as ease of patient understanding, and time constraints, and as of the time of this publication, there is no body of literature addressing these considerations.

A key concern when implementing interventions in clinics is the limited funding and resources available [10]. This issue can constrain the variety of interventions that can be implemented, highlighting the importance of understanding the common barriers faced by free clinics in addressing FI. Furthermore, the role of free clinics as a safety net for at-risk populations emphasizes the importance of identifying the common barriers patients face in using these interventions. Identifying barriers across multiple free clinics can enable better tailored future FI interventions.

Additionally, the topic of screening and addressing FI in free clinics has been limited to results and conclusions drawn from individual clinics. Broadly characterizing this field can provide valuable insights into the differences among implemented food insecurity interventions, enabling future efforts to explore new approaches or avoid similar challenges.

Our aim is to examine food insecurity screenings and interventions at free clinics across the U.S. In this review, we will provide demographic information and screening statistics of food insecurity interventions, such as the type of screening form used, the number of patients screened, the percentage of FI patients. Additionally, we will discuss information about the study’s primary aim, and how it relates to the provided FI intervention. Finally, we will discuss barriers that studies identified in implementing FI interventions.

## METHODS

Our protocol was drafted using the PRISMA Extension for Scoping Reviews (PRISMA-ScR) checklist. To be considered for inclusion in our scoping review, studies needed to implement a FI screening and intervention program in a free clinic within the United States specifically due to the unique nature of the healthcare system. Studies could address additional social determinants of health such as or chronic conditions such as (e.g. obesity, Type 2 Diabetes Mellitus), but had to include FI screenings and interventions. Studies had to be published in an English, peer-reviewed journal. Studies on the implementation of FI programs in nonprofit and for-profit hospitals, and federally qualified health centers were excluded, as there is already significant research on FI interventions. Additionally, studies only including FI screening, but not FI interventions were excluded. Studies published in student journals, editorials, letters, commentaries, and other review papers were excluded to ensure that only peer-reviewed primary research was considered. Studies including the pediatric population were excluded.

We conducted searches in the following databases: PubMed, Web of Science, Cochrane, Academic Search Complete via EBSCO and Google Scholar. The search strategy for this review was built by author G.R, W.Z, alongside an experienced medical research librarian (E.S) and refined and conducted by E.S. The final search was conducted on August 8, 2024.

Search results were screened in Rayyan. Screening was conducted at the title, abstract, and full-text levels. Screening at the title, abstract and full text levels were conducted independently by three authors (GR, WZ, DW). Each reference was screened by two of the three stated authors. Exclusion was contingent on agreement between both reviewers. During full-text screening, journals were only included if they were present within the Eastern Virginia Medical School’s database of journals. After full-text screening, differences in opinion (n = 12) were resolved through discussion by all three authors.

Three authors (W.Z, D.W, R.F) manually extract and record relevant and consistent information from the final included studies (n = 5). Additional data were not obtained or confirmed with investigators.

The variables collected included: title of study, authors, year, location, aims, FI screening tool, sample size, patient mean age, standard deviation (SD) and range, FI prevalence, intervention, barriers reported, and intervention limitations. Patient mean age, SD and range refers to patients who were screened by the FI screening tool during intake. Sample size was defined as the total number of patients screened in the clinic for FI. FI prevalence was defined as the percentage of patients who were screened and tested positive by the questionnaires used. The type of intervention refers to the programs and strategies implemented by free clinics to address a positive food insecurity screening. Barriers reported refer to issues faced by FI patients in utilizing the interventions provided by the free clinic. Intervention limitations refers to issues reported by the authors in addressing positive FI screening results. If information was not provided by the authors, N/A was used as a placeholder.

Data including title, year of publication, location of the clinic(s), aims of the study, FI screening tool utilized, sample size of the study pool, mean age of the patient population, FI prevalence in the patient population, total patients screened for FI, interventions, barriers identified contributing to FI, and barriers identified limiting intervention effectiveness were extracted from the 5 included studies, and summarized by three authors, W.Z, D.M, and R.F. Descriptive data was directly collected for interventions to combat FI, barriers identified contributing to FI, and barriers identified limiting intervention effectiveness. FI prevalence in Dembar et al. (2020) and Nguyen et al. (2022) was manually calculated as a percentage by authors G.R and W.Z [11, 12].

The initial database search yielded 958 studies. Title screenings were conducted of the 958 initial studies. Each title was assessed for mentions of food insecurity and/or free clinics. 26 studies passed to the abstract screening level. 2 studies were duplicates and were removed. The remaining 24 studies were assessed for screening and addressing food insecurity at free clinics. A total of 9 full text studies met criteria. During full text screening, only studies that had contained both screenings and direct interventions for FI patients and were in peer-reviewed journals were included in this review. 5 studies were included in this scoping review for analysis.

## Results

The goals of the study, questionnaire details, percentage of food insecure patients, identified barriers, and resources provided, as well as the study year, location (city and state), sample size, age of patients, and descriptive statistics are provided in Table 1. Descriptive statistics include FI prevalence (%), patients screened for FI (%), interventions implemented, barriers identified contributing to FI, and barriers identified limiting intervention effectiveness.

All studies were published between 2016 and 2024, and took place in four different cities across the United States, New York, Milwaukee, Nashville and San Diego. Two studies took place in San Diego. The number of patients experiencing food insecurity varied widely between clinics in the five studies, between 18.5 to 85.7%. Even though FI prevalence was always higher than the national average, 12.8%, there was a lack of data analyzing the effectiveness of FI interventions at free clinics, except for Dembar et al. (2020) [11 12]. In three of the five studies, the 6 item HFSS was used to assess food insecurity [11, 14, 15]. Nguyen (2022) used one question from the USDA food security survey to determine food security among patients [12].

Miller et al. (2024) assessed food insecurity alongside other social determinants of health in their patients through a custom 12 item redcap survey identifying social determinants of health [9]. (Appendix 2) Nguyen et al. (2022), Smith et al. (2016), and Toma et al. (2020), reported screening rates of 80.3, 92.5, and 95.9 respectively [12, 14, 15] All studies except for Miller et al. (2024) distributed food directly to patients [9]. The method of food distributions varied between studies. Dembar et al. (2020) provided grocery deliveries, Nguyen et al. (2022) and Toma et al. (2020) utilized in clinic food pantries and Smith et al. (2016) provided onsite food boxes [11,12 14, 14]. Two programs, Miller et al. (2024) and Smith et al. (2016) aided patients in accessing SNAP, WIC and food pantries in the area, and three provided referrals [9, 11, 14].

Dembar et al. (2020) and Toma et al. (2020) identified barriers that contributed to food insecurity amongst patients in general [11, 15]. Dembar et al. (2020) cited that patients had limited time due to work schedules, lack of transportation to resources, lack of identification, unstable, consistent housing arrangements, limited financial resources, insufficient support, inability to cook provided items, and food expiring [11]. Toma et al. (2020) stated that transportation constraints, embarrassment, fear of rejection and documentation status may contribute to patients not receiving appropriate aid [15]. Nguyen et al. (2022) outlined difficulties with food distribution, stating that food waste was produced by lack of food supply utilization due to cultural, procedural, and physical barriers [12].

## DISCUSSION

### Locations

Most of the clinics listed in the included studies were based in various cities and/or campuses throughout the country. However, Smith et al. (2016) and Toma et al. (2022) implemented Fl interventions in San Diego through the University of California, San Diego Student Run Free Clinic project at different time points.Both programs screened patients for FI based on the 6-item USDA Food Security Survey, and provided food on-site [14, 15].

Smith et al (2016) provided boxes of food for FI patients specifically, and a referral program. This program included onsite assistance in SNAP enrollment and, if the patient also had concomitant diabetes, eligibility to receive diabetes-appropriate foods at the clinic, through collaboration with community partners [14]. If the patient did not have concomitant diabetes, then off-site referrals to local food pantries were provided.

The intervention utilized in Toma et al (2022) was similar to that of Smith et al. (2015), as they provided an on-site food pantry via partnering with community food banks [14, 15]. However, in this case, all patients, regardless of their food security, were eligible for onsite food distribution. However, there were no referrals provided to patients.

Smith et al (2015) found that 318 of the 430 patients (74.0%) screened for FI were FI positive. Of these 318, 201 (63.2%) received food onsite, 66 (20.8%) received food from an off-site pantry, and 64 (20.1%) enrolled in SNAP benefits [14]. Toma et al (2022) included a total of 409 patients that participated in the program. Prior to installation of the intervention, 314 out of 409 (76.8%) of the patients screened positive for FI. This FI positive patient population decreased to 240 out of 409 (58.7%) at the end of the 3.4-year survey study [15].

Given the similarities in location, free clinic programs, and patient populations, these two studies give insight on the potential effectiveness of different FI interventions. The implementation of an onsite food pantry open to any patient regardless of FI screening results, as in Toma et al (2022), was able to lower FI within the given patient population. This decrease, while its statistical significance is unknown, over the 3.4-year study could suggest implementation of an all-inclusive food pantry could be beneficial in improving food insecurity rates within the San Diego Health System. As other studies that provided food pantries in free clinics did not evaluate changes in FI status among its patients, it is difficult to determine whether an all-inclusive food pantry are equally effective in every free clinic.

Though Smith et al. (2015) was unable to screen for FI following longstanding implementation of the program, the similarity of FI positive patients between Smith et al. (2015) and Toma et al. (2022) (74% and 76% respectively) could imply that the referral program in Smith et al. (2015) was not useful for patients [14, 15].

This is supported by the fact that data from Toma et al. (2022) showed that the barriers patients faced in addressing their FI was a lack of transportation, which would have limited the usage of the referral program in Smith et al. (2015) [14, 15]. Indeed, Kakarala et al. (2023) showed a significant, positive correlation between FI and transportation insecurity [16]. As such, it could have been that access to transportation could potentially improve access to FI resources, and effectiveness of FI referrals provided in clinics.

There are significant limitations that reduce the applicability of the Smith et al. (2015) Toma et al. (2022)’s interventions to other free clinics. These major barriers would be the availability of local community partners [14, 15]. One study, Cullen et al. (2020) found that patients geographical access to FI resources is one of the single most important factors in determining qualitative efficacy of resource referrals[17].

Overall, regardless of the intervention, both studies suggest that any intervention that attempts to mitigate Fl in its vulnerable patient population provides some level of upside and effectiveness.

### Food Questionnaires

Studies in this review utilized an already existing USDA food security survey, certain components of the USDA survey, an adapted model based on the USDA survey, or a custom survey. These surveys, administered as questionnaires to patients, revealed variable percentages of food insecurity amongst patients in each of the clinics, with a range from 18.5–85.7%. The percentage of FI patients was higher than the national average of FI 12.8% [18]. The presence of high levels of FI relative to the national average suggests that student-run free clinics should routinely screen for food insecurity.

Miller et al. (2024) indicated that surveys were administered verbally by trained staff members [9]. Smith et. al., (2016) indicated that surveys were administered through written forms that were filled out by patients or their family members, with trained staff available to verbally administer them if needed [14]. While the percentage of patients assessed for FI status in Miller et al. (2024) was not provided, Smith et al. (2016) was able to assess 92.5% of patients attending clinic for FI status [9]. Therefore, it is possible to implement said surveys in clinics with a variety of different staffing and procedural needs while still assessing for food insecurity. However, some methods, such as phone surveys, can result in barriers that prevent eligible patients from being accounted for as many individuals may have limited access to phones, or may use prepaid phones with limited minutes [11, 14, 19]

In Miller et al., the percentage of food insecure patients was significantly less than the other studies [9]. This could be possible due to the variance in the screening process, as this study used a clinic-generated redcap form as opposed to other studies which used previously established, validated and sensitive food insecurity screening forms [20].

Three studies reported the percentages of total patients screened for Fl. Nguyen et al. (2022) reported screening 132 out of 132 total patients (100%), Smith et al. reported screening 430 out of 463 total patients (92.5%), and Toma et al. reported screening 513 out of 535 total patients (95.9%) [12, 14, 15]. Factors preventing all patients from being screened could have included patient worries regarding confidentiality, lack of training among clinicians, and time constraints [20].

### Identified Barriers

FI patients faced varied barriers preventing them from accessing food resources, and several barriers have been identified. Across the reviewed studies, patients faced logistical issues when trying to address their FI status, such as the inability to properly store or prepare food and/or insufficient amount of food provided by pantries [14, 15]

Dembar et. al. (2020) referred patients to external resources soup kitchens and food pantries. Prior research conducted by Dembar et al. (2020) through EHHOP surveys showed that psychosocial barriers such as difficult work schedules, mobility problems, lack of identification, unstable housing arrangements, limited financial resources, insufficient food provisions, and incompatible types of food provisions with their dietary needs reduced the effectiveness of these referrals. Dembar et. al. (2020) indicated that their barrier informed referral approach was helpful in many patients, but that housing instability and poor clinical follow-up continued to limit its efficacy [11]. Toma et. al. (2022) also referred patients to external resources. Similarly to Dembar et al. (2020), they found that documentation status (i.e, identification status), transportation constraints (i.e, mobility problems) contributed to reduced utilization of referred resources [11, 15].

The presence of several common barriers across these studies highlights the structural and systemic nature of these barriers, emphasizing the need for interventions that address issues such as improving access or bypassing the need for transportation, offering more flexible service hours, or providing alternative support for those without documentation at a baseline.

There were unique barriers identified in each study. Smith et al. (2016) identified that patients experienced technological issues when they applied for SNAP benefits [14]. Toma et al. (2022) found that embarrassment and fears of rejection also contributed to reduced utilization of food pantry referrals [15].

Miller et. al. (2024) found unique barriers through evaluating other social determinants of health. Specifically, they found that Fl is associated with other financial stresses such as housing instability (r = 0.55), and medication (r = 0.53) because they are competing monetary priorities among patients. Additionally, they reported that mental health needs were related to food insecurity (r = .42) [9]. Due to this significant correlation, it can potentially make it difficult for interventions specifically focused on addressing FI to be effective without addressing these other social determinants of health. Overall, the presence of unique barriers indicates that free clinics should design interventions that are barrier informed.

Some studies utilized in-clinic food pantries as their main intervention [12, 15]. This intervention has benefits that directly address the barriers discussed above. Toma et. al. (2022), found that with this type of intervention, there was a decrease in the level of stigma and shame felt by patients during visits, and increased satisfaction with food items [15]. Additionally, Nguyen et al. (2022) found that portioning out available food to each patient reduced the effect of the social barriers, such as not wanting to take a significant amount of food from a communal supply [12].

However, in-clinic food pantries have their own drawbacks as well. Nguyen et. al. (2022) and Toma et. al. (2022) identified that cultural barriers, procedural disturbance from clinic operations and physical barriers can impede distribution of foods in clinic, and logistical problems arise in balancing inventory and preventing wastage during stocking when allowing patients to freely choose provisions [12, 15]. To overcome these additional barriers, Toma et. al. (2022) suggest increasing nutritional guidance would make that method of food distribution more effective [15].

### Interventions

A variety of methods were used to reduce food insecurity within patient populations. However, of the studies discussed in this study, only two evaluated the effectiveness of the intervention. Dembar et al. (2020), found that their patient-barrier-informed interventions decreased FI in the screened patient population from 85.7–50% [11]. Toma et al. (2022) found their all-inclusive food pantry decreased FI in the screen patient population from 76.8–58.7% [15]. Both of these approaches resulted in removing or mitigating the detrimental effects of relevant barriers to food-insecure patients.

Dembar et al. (2020) accounted for relevant patient-specific difficulties in accessing food by making referrals with food pantries that had the fewest patient-identified-barriers, or by providing grocery deliveries for a week [11]. Toma et al. (2020) provided an on-site food party that was accessible to all patients regardless of food insecurity status [15]. Both of these interventions targeted transportation barriers. Sharareh et. al. (2022) indicate that transportation is an important barrier to accessing food among many people, since availability of transportation can affect whether or not it is possible or affordable for people to acquire food [21]. The result of the interventions done by Dembar et al. and Toma et al. was successful alleviation of transportation as a barrier to accessing food for food insecure patients [11, 15].

In addition to alleviating transportation barriers, Dembar et al. and Toma et al. may have also reduced the salience of social barriers to accessing food through their interventions [11, 15]. Dembar et al. had specially-trained staff who accommodated the patients’ needs, which probably made the provided assistance more appealing and actionable to patients than a traditional list of available resources would [11,. Toma et al. provided patients with a means to access food in a way that patients would likely find significantly less stigmatizing or embarrassing than going to a food pantry or other charity organization [15]. These social factors likely had a significant influence on improving the food insecurity status of relevant patients.

### Intervention Limitations

Many of the studies analyzed held common threads in terms of FI intervention limitations. There was significant recall bias in all studies except for Miller et al. (2024) [9]. A positive Fl identifier solely depended on results listed the given surveys, including but not limited to the 6-item USDA Food Security Survey and the USDA’s Household Food Security Survey. Self-reporting in these surveys could have resulted in exaggerated or dampened responses due to a number of factors. Toma et al. (2024) suggests that exaggerated responses might be due to fear of omission from food distributions or referrals [15]. Moreover, Smith et. al. (2016) hypothesized that patients may not indicate that they are food insecure during surveys due to shame or preference to not discuss their food insecurity with a healthcare provider. This barrier highlighted by Smith et al. (2016) is a common thread that many healthcare providers experience. In a questionnaire completed by over 2800 healthcare providers, including but not limited to medical doctors, advanced practice providers, and registered dietitians [14]. Approximately 851 providers (30.4%) experienced patient resistance in discussing FI status and 479 providers (17.1%) experienced patient resistance in discussing community food programs [22].

Another limitation listed by the studies was inability to measure the effectiveness of the administered interventions. From the five studies included in this scoping review, only Toma et al. (2024) was able to screen for FI following administration of the program, thereby providing a measure of comparison between pre- and post- Fl intervention administration. Though the remaining studies also screened for Fl and provided an intervention (i.e. community partner referral program, on-site food distribution), they did not provide FI screening values following program administration which limits conclusions which can be drawn.

### Future Directions

Future studies investigating the implementation of programs targeting food insecurity should focus on an evaluation of the effectiveness of the programs. Most studies to date have assessed measurements of food insecurity pre and post intervention [15].

It is essential to identify and strategize ways to overcome the common barriers found across studies, such as patient recall bias in completed FI surveys, in order to successfully implement food insecurity interventions, and to promote food security in general among patients utilizing free clinics.

Future studies could also assess the implementation of other FI screening assessments in identifying FI patients. For example, Nguyen et al. (2022) found that utilization of the Food Quality Assessment Survey underrepresented the degree of FI within their student-run free clinic. Instead, through utilizing other methods, such as the Hunger Vital Sign, they were able to better identify FI within their patient population. Other studies found similar results as well [12]. When compared to a 18-item Household Food Security Survey Model, the Hunger Vital Sign identified 26% more Fl-positive patients in a given cohort of 5039 respondents [23]. Again, this suggests that utilization of other screening procedures might allow for a greater range of assessment in FI within a patient population.

Lastly, future studies could work to assess the efficacy of implemented Fl interventions on various patient health outcomes. The relationship between FI and health outcomes is still vaguely understood. With some literature suggesting that physical limitations result in decreased food security while others suggesting the opposite, this poor understanding of the relationship between food security and overall patient health could be further explored through the assessment of FI interventions in various SRFCs [24, 25].

The limitations for this scoping review came down to a lack of substantive research in this field. Only five studies met screening criteria producing a very small sample size for analysis. Of these, only two measured changes in FI status of patients. This made it difficult to do a robust comparison of FI interventions across free clinics.

Additionally, there was significant variation in the percentage of patients who screened positive for food insecurity in each program. In Wisconsin, there is no evidence that food insecurity is associated with population density or poverty by zip code, therefore these factors may not contribute significantly to the variation in other areas of the USA [26]. These differences may be due to differences in demographics for the populations in the areas around each clinic. For example, Toma et. al. had a population of 99.8% adult hispanic persons, with 78.6% food insecurity, Nguyen et. al. had a population of only 53.8% hispanic persons, with 80.7% being food insecure, and Miller et. al. had only 31.9% hispanic persons, yet only 18.5% of them were found to be food insecure [9, 12, 15]. This suggests that further evaluation of food insecurity by demographic may be warranted to better evaluate the effectiveness of interventions on relevant demographic populations. Alternatively, this variation may be due to inconsistent eligibility requirements for services provided by the different clinics.

Screening methods for food insecurity in the reviewed studies were largely derived from the United States Department of Agriculture. While this indicates that the USDA survey is contributing to standardization in food insecurity assessment, it provides limited room for assessment of its effectiveness relative to other screening methods. Toma et. al. (2022) mentions various other validated screening methods that could be utilized, including a 1-Item Hunger Screening, a 1-Item Question Screening from the SEEK screener, and the USDA-FSS module variations with 8-item, 10-item, and 18-item surveys. Patient responses to self-report surveys may be influenced by social factors and recall bias, which can lead to underreporting of food insecurity [27]. By utilizing and comparing the effectiveness of these other validated surveys in these programs, it may be possible to identify assessment methods that are better at minimizing the effect of these limitations, which will make it easier to establish best practices.

Although Miller et. al. (2024) included repeated surveys to assess for effectiveness of provided resources, they found that a 30 day interval between surveys was too fast for patients to effectively utilize the resources. Further, their follow-up survey focused on resource usefulness, not effectiveness. Future studies should follow up with patients to re-assess them for food insecurity, over a longer interval of time, in order to assess the effectiveness of interventions, which can additionally contribute to the formation of best practices [9].

Student run clinics across the country should administer a food insecurity questionnaire to assess patients’ food insecurity given the high prevalence of food insecurity in free clinics relative to the national average [18]. Clinics should aim to provide referrals and/or direct food supplies to patients if possible and should work with patients to create individualized plans to determine how to best increase access to food. Referrals to external resources should focus on alleviation of housing instability and medication needs as well as food insecurity, since they are often co-occurring financial stresses that can reduce the effectiveness of interventions with a more limited scope. In-clinic food pantries should strongly consider portioning their food to their patients to alleviate logistical concerns, and to provide nutritional guidance to enhance the satisfaction that patients receive from portions that they select. Finally, future studies should consider the use of other validated screening methods and should evaluate post-intervention effectiveness for sake of comparison. This will make it possible to more effectively assess for and relieve food insecurity in relevant populations, whether by avoiding known barriers to the effectiveness of interventions, or by contributing to the formation of more evidence-based best practices.

## Figures and Tables

**Figure 1:**
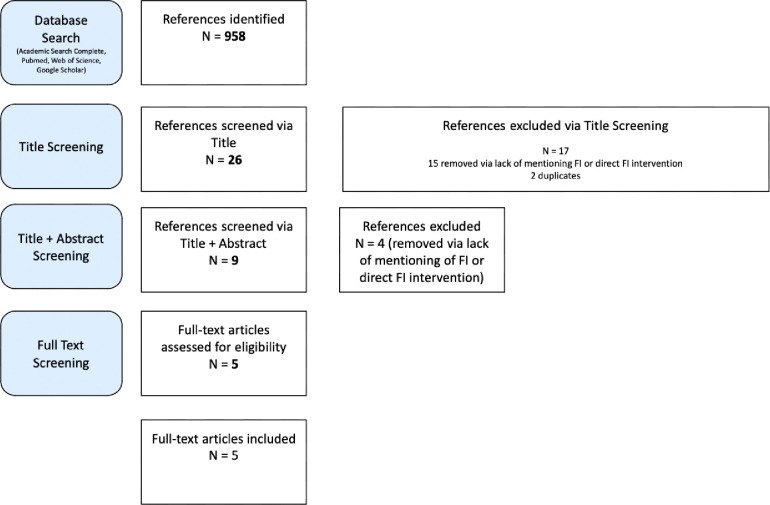
Study Screening Flowchart

**Table 1:** Compiled Results of FI Screenings and Interventions at Free Clinics in the United States

Title	Year	Author	Location	Aims	FI Screening Tool	Sample Size (N=)	Mean Age ± SD (yrs.), Range (yrs.)	FI Prevalence (%)*	Patients Screened for FI (%)	Intervention	Barriers identified contributing to FI	Barriers identified limiting intervention effectiveness
Reducing food insecurity through personalized interventions at the East Harlem Health Outreach Partnership	2020	Dembar et al.	East Harlem New York, NY	Assess effectiveness of individualized FI interventions	1. HVS 2. Six item HFSS	21	Mean Age = 47 ± 11.1 Range = 25–74	85.7 to 50	N/A	1. Food pantry or kitchen referral 2. Barrier-Informed individualized interventions 3. Grocery delivery to home	1. work scheduling 2. transportation 3. lack of identification, 4. unstable housing arrangements 5. limited financial resources 6. insufficient support 7. lack of ability to cook food 8. Food perished	N/A
Social determinants of health correlations and resource usefulness at a Milwaukee free clinic for uninsured individuals: A cross-sectional study	2024	Miller et al.	Milwaukee, WI	Assess correlations between social determinants of health needs and patient determined longitudinal psychosocial resource usefulness	12-item custom SDOH REDCap Survey	238	Mean Age = 48 SD N/A Range N/A	18.5	N/A	1. Food Pantries and Farmer's Market Locations 2. Food Delivery for Seniors programs 3. SNAP and WIC	N/A	N/A
Experiences with a Student-Run, In-Clinic Food Donation Program for Uninsured Patients in Nashville	2022	Nguyen et al.	Nashville, TN	Describe experiences with an in-clinic free food pantry for uninsured patients	1. 2 question intake form 2. Food Quality Assessment Survey	132	N/A	65.4 to 69.2	100%	In clinic food pantry	N/A	1.Cultural Barriers 2. Procedural Disturbance 3.Physical barriers
Implementation of a food insecurity screening and referral program in student-run free clinics in San Diego, California	2016	Smith et al.	San Diego, CA	Record and address FI prevalence through a screening program	Six item HFSS	430	Mean Age = 51.2, ± 11.4 Range N/A	74	92.5	1 Onsite food box distribution 2. Food pantry referrals 3. On site SNAP enrollment	N/A	
Implementation of an On-Site Food Prescription Project to Address Food Insecurity in Multiple Free Clinic Sites Serving an Adult Latinx Population	2022	Toma et al.	San Diego, CA	Assess effects of the implementation of the Food Prescription Project on FI	Six item HFSS	535	N/A	76.8	95.9	In clinic food pantry	1. transportation constraints, 2. embarrassment, 3.fears of rejection 4. documentation status	N/A

HVS = Hunger Vital Sign

HFSS = Household food security survey

SNAP = Supplemental Nutrition Assistance Program

WIC = Women, infants, and children program

* Prevalence was calculated by the percentage of patients answering “often” or “sometimes” to the questions “In the past year, I worried whether my food would run out before I got money to buy more” or “In the past year, the food I bought just did not last, and I did not have money to get more”
